# Expression of selenium-independent glutathione peroxidase 5 (GPx5) in the epididymis of Small Tail Han sheep

**DOI:** 10.5713/ajas.18.0015

**Published:** 2018-04-12

**Authors:** Ruilan Li, Xiaomei Fan, Tong Zhang, Huizi Song, Xiaona Bian, Rile Nai, Jinquan Li, Jiaxin Zhang

**Affiliations:** 1 College of Animal Science, Inner Mongolia Agricultural University, Hohhot, Inner Mongolia 010018, China; 2 Key Laboratory of Animal Genetics, Breeding and Reproduction, Inner Mongolia Autonomous Region, Hohhot 010018, China; 3 Basic Medical College, Inner Mongolia Medical University, Hohhot, Inner Mongolia 010110, China; 4 Key Laboratory of Mutton Sheep Genetics and Breeding, Ministry of Agriculture, Hohhot 010018, China

**Keywords:** Epididymis, Sperm, GPx5, Oxidative, Reactive Oxygen Species

## Abstract

**Objective:**

Selenium-independent glutathione peroxidase (GPx5) is specifically expressed in the mammalian epididymis and plays an important role in protecting sperm from reactive oxygen species and lipid peroxidation damage. This study investigates GPx5 expression in the epididymis of Small Tail Han sheep.

**Methods:**

GPx5 expression was studied in three age groups: lamb (2 to 3 months), young (8 to 10 months), and adult (18 to 24 months). The epididymis of each age group divided into caput, corpus and cauda, respectively. Analysis the expression quantity of GPx5 in epididymis and testis by real-time fluorescent quantitative polymerase chain reaction and Western blot. Finally, GPx5 protein locating in the epididymis by immunohistochemical.

**Results:**

The results demonstrate that in the lamb group, the GPx5 mRNA, but not protein, can be detected. GPx5 mRNA and expressed protein were detected in both the young and adult groups. Moreover, both the mRNA and protein levels of GPx5 were significantly higher in the young group than in other two groups. When the different segments of epididymis were investigated, GPx5 mRNA was expressed in each segment of epididymis regardless of age. Additionally, the mRNA level in the caput was significantly higher than that in corpus and cauda within same age group. The GPx5 protein was in the epithelial cells’ cytoplasm. However, GPx5 mRNA and protein were not detected in the testis.

**Conclusion:**

These results suggest that GPx5 is mainly expressed in the epididymis of Small Tail Han sheep, and that the expression level of GPx5 is associated with age. Additionally, GPx5 was primarily expressed in the epithelial cells of the caput. Taken together, these studies indicate that GPx5 is expressed in the epididymis in all age grades.

## INTRODUCTION

The epididymis plays an important role in reproduction for mammalian species as it is the organ responsible for spermatozoa maturation and storage. Before entering the epididymis, spermatozoa have already completed their differentiated morphological change, but have not yet acquired motility and fertilization potential. Spermatozoa progressively acquire their potential to fertilize oocytes during epididymal transportation, depending on the luminal environment and epididymal duct created by the epididymis epithelium. During their progression through the epididymal duct, spermatozoa plasma membranes undergo many modifications [1].

A suitable level of reactive oxygen species (ROS) is a key factor for normal sperm physiology in maturation and capacitation [2]. ROS regulates the disulfhydryl formation that contributes to the packaging of the nucleus [3]. Excess ROS in the epididymal duct can cause oxidative stress, which damages sperm DNA and macromolecular structure [4,5]. Moreover, the high poly-unsaturated fatty acid content of the spermatozoa plasma membrane becomes more susceptible to oxidative stress after most of their cytoplasm is lost during the process of spermatogenesis [2,6]. Therefore, sustaining a normal level of ROS is a key factor in determining normal sperm function. Epididymal epithelial cells offer protection against oxidative stress through ROS scavengers such as catalase, superoxide dismutase, and glutathione peroxidase (GPx) [2]. GPxs have a particularly important role, as several GPx proteins reside in the spermatozoa plasma membrane, and are found in close physical proximity to spermatozoa as they transit through the excurrent duct system. Additionally, GPxs are responsible for eliminating a variety of substrates besides H_2_O_2_, including organic peroxides and peroxynitrite anion [2,7,8].

The GPx5 known as epididymis GPx and is expressed only within the epididymis of certain mammals, including mice [9], rats [10], dogs [11], boars [12]. GPx5 is different from other GPxs in that it lacks a selenocysteine residue, but still retains its antioxidant properties. Lipid peroxidation generates highly reactive malonaldehyde that damages sperm DNA in the absence of GPx5 [13]. GPx5 is secreted by the epididymis and comprises more than 95% of the total GPxs present in the epididymis fluid [14]. GPx5 combines with sperm through the epididymosome during epididymis transportation [15]. Mating wild-type females with GPx5-knockout male mice resulted in a higher rate of miscarriage and embryonic defects because of sperm DNA damaged. This demonstrates that GPx5 has an essential role in protecting spermatozoa from oxidative stress damage [13].

To our knowledge, no detailed GPx5 expression patterns in sheep epididymis cells have been previously reported. In this study, the expression of GPx5 in Small Tail Han sheep epididymis was investigated. The expression of GPx5 in sheep epididymis can further our understanding of the antioxidative mechanisms in sheep and other mammals.

## MATERIALS AND METHODS

### Samples

**Figure 1:**
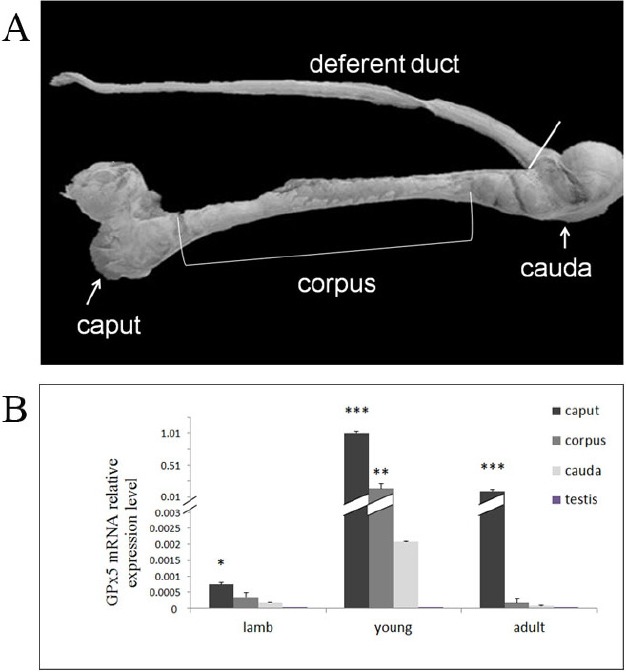
(A) Different segments of sheep epididymis from which tissue extracts were prepared to study selenium-independent glutathione peroxidase (GPx5) distribution. (B) The quantity of expressed GPx5 mRNA in different segments of epididymis and testis of sheep at different ages. The expression profile of the relative expression levels of GPx5 mRNA in young and adult sheep compared to lambs within the segments of epididymis. * p<0.05; ** p<0.01; *** p<0.001.

The testis and epididymis of three different age groups—lamb (2 to 3 months), young (8 to 10 months), and adult (18 to 24 months)—were obtained from local slaughterhouse. The procedure for collecting tissues samples conformed to relevant guidelines and regulations issued from the Ministry of Agriculture of the People’s Republic of China. Samples in each group were collected from six sheep. After collecting, samples (Figure 1A) were immediately placed on ice and dissected. Tissues were snap frozen in liquid nitrogen and stored at −80°C.

### RNA extraction from sheep testis and epididymis with quantitative reverse transcription–polymerase chain reaction analysis

Snap-frozen pieces of testicular and epididymis tissue from the three sheep groups were ground with a mortar. Total RNA was isolated with TRNzol (TIANGEN, Beijing, China) according to manufacturer’s instructions. RNA integrity was assessed by agarose gel electrophoresis and then reverse transcribed using the PrimeScript RT Master Mix kit (Tarkara, Tokyo, Japan). GPx5 and the housekeeping gene β-actin were amplified by standard polymerase chain reaction (PCR) using the Taq PCR Mastermix kit (TIANGEN, Chaina) and analyzed on agarose gel to assess the presence of genomic DNA in each extract. Primer sequences and specific PCR conditions for all targeted candidates are presented in Table 1. For standard PCR amplification, the general reaction steps were as follows: initial denaturation/enzyme activation at 95°C for 5 min followed by 35 cycles of denaturation at 95°C for 30 s, annealing at 62°C for 30 s, and extension at 72°C for10 min. The amplified PCR products were electrophoresed on agarose gel. SYBR Premix Ex TaqTMII (Tarkara, Japan) was used for GPx5 transcription relative quantification per the manufacturer’s instructions. The reaction conditions were as follows: initial denaturation/enzyme activation step at 95°C for 10 min, and 40 PCR cycles as follows: 5 s denaturation at 95°C, 30 s annealing (see Table 1 for temperatures), and 30 s elongation at 72°C. Expression data were normalized with the housekeeping gene (β-actin) based on the relative quantification approach proposed in the method for 2^−ΔΔct^, (ΔCt = Ct [objective]–Ct[negative]); ΔΔCt = (ΔCt[other tissues]–ΔCt[young sheep epididymis caput]). All PCR products were analyzed on agarose gel.

### Protein extraction from sheep testis and epididymis with immunoblot analysis

Samples of sheep testis tissue and epididymis segments (caput, corpus, and cauda) were prepared by homogenizing in cell lysis buffer for immunoblot and immunoprecipitation analysis with 0.1 volume of PMSF (1 mM) and centrifuged at 15,000 r for 25 min at 4°C. Concentration of protein was assayed using the bicinchoninic acid (Baitaike, Beijing, China) kit based according to manufacturer’s instructions (Baitaike, China), using bovine serum albumin (BSA) as standard, and storing at −20°C until used.

Protein was separated on 12% sodium dodecyl sulfate–polyacrylamide gel electrophoresis polypropylene gel on the basis of equivalent quantity of proteins per lane (30 μg), and then transferred to polyvinylidene fluoride (PVDF) membrane (Thermo Fisher, Waltham, MA, USA) using the semi-dry method membrane blotter system. Following PVDF, membranes were blocked for 1 h at room temperature in 5% (w/v) skim milk and incubated with purified rabbit anti-GPx5 (1:500) diluted in blocking solution overnight at 4°C. Membranes were then washed in PBS with 1% Triton X-100 and placed with goat anti-rabbit immunoglobulin G (IgG) secondary antibody coupled with horseradish peroxidase (HRP) (dilution 1:1,000; Bossion) for 1 h at room temperature. Enhanced chemiluminescence was used as the peroxidase substrate (Thermo Fisher, USA). Other membranes were blotted with 1:1,000 diluted mouse anti-β-actin (Abcam, Cambridge, MA, USA) for the primary antibody and 1:5,000 diluted goat anti-mouse horseradish peroxidase coupled as the secondary antibody under the same conditions.

### Immunohistochemistry

GPx5 detection was performed on the different epididymis segments. The tissues were fixed with 4% formaldehyde in PBS for 48 h at 4°C. After dehydration, transparentizing tissues were embedded in paraffin, and 4 μm thin sections were prepared on glass slides. After de-parafinization and rehydration, sections were soaked in 3% hydrogen peroxide in dd-H_2_O for 20 min to neutralize endogenous peroxidases. Sections subjected to antigen retrieval using 0.01 mM citrate buffer and blocked using 5% BSA for 30 min, were incubated with anti-GPx5 (IgG) antibody (1:250) in 5% BSA overnight at 4°C followed by 4 washes using PBS (5 minutes each), then incubated with HRP-conjugated goat anti-rabbit IgG for 1 h at room temperature. Signal was revealed with DAB kit (MAIXIN_Bio, Fujian, China). Nuclei were then stained by hematoxylin. BSA was used as negative control. Protein GPx5 were stained brown in tissue and the nuclei were stained blue.

## RESULTS

### Expression of GPx5 mRNA in sheep epididymis and testis

Quantitative reverse transcription (qRT)-PCR analysis on extracts from the caput, corpus, and cauda revealed that GPx5 mRNA was expressed in every age group. The mRNA levels in the different segments of epididymis was higher in the young group than in the lamb group (p<0.001) and the adult group (p<0.01) (Figure 1B). The expression levels of GPx5 mRNA in the caput was significantly higher than in other segments in the lamb group (p<0.05), while the level of GPx5 mRNA in the caput was much higher than in the other segments in the young group and adult group (p<0.001) (Figure 1B). However, GPx5 mRNA was hardly detected in the testis (Figure 1B).

### Identification of GPx5 protein in sheep epididymis

**Figure 2:**
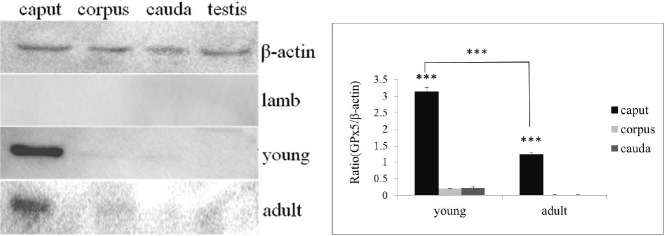
Immunoblot detection of glutathione peroxidase (GPx5) in protein extracts from segments of the caput, corpus, and cauda epididymis and testis. GPx5 protein was detected in different segments of the epididymis and testis of sheep with different ages (the different age groups are presented on the right).

Immunoblot analysis of the GPx5 protein in the sheep epididymis was not also detectable in the epididymis from the lamb group. In the young group, GPx5 protein was detected in each section except for the testis. Expression of GPx5 protein in the caput was higher than in the corpus and the cauda (p<0.001). In adult sheep, GPx5 protein was detectable in the caput, but not in the other segments of the epididymis or testis. The level of GPx5 protein was greatly decreased in the caput of the adult group compared to the young group (p<0.001) (Figure 2).

### Immunohistochemistry of GPx5 in sheep epididymis

**Figure 3:**
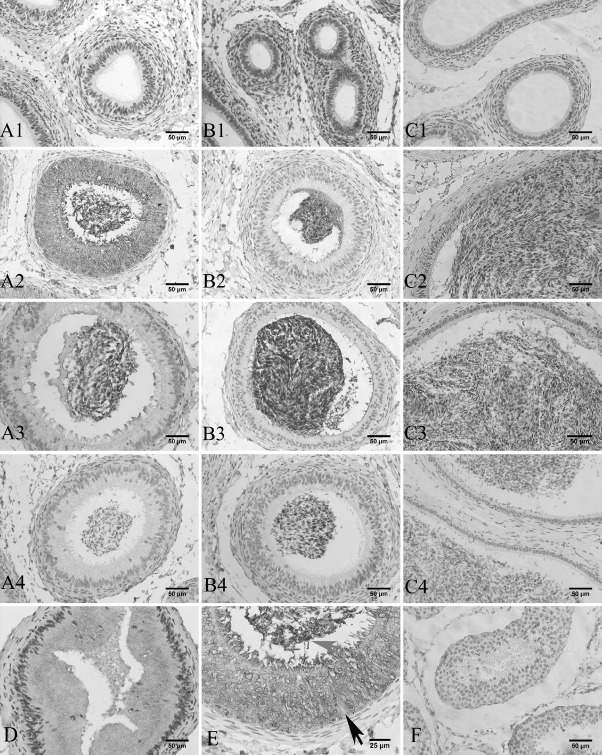
Immunohistochemical detection of epididymis. Brown staining is a positive signal, blue staining is the cell nucleus (Bar = 50 μm). A1, B1, and C1 denote the caput, corpus, and cauda in the treatment groups of lamb and young epididymis, respectively. A2, B2, and C2 denote the caput, corpus, and cauda in the respective treatment groups for adult sheep epididymis. A3, B3, and C3 refer to the caput, corpus, and cauda in the respective treatment groups. A4, B4, and C4 refer to the corresponding negative controls using bovine serum albumin. D denotes the proximal of the caput. E denotes the distal of the caput in the young sheep (Bar = 25 μm). Black arrow indicates cell nucleus; gray arrow indicates sperm. F denotes the testis treatment group.

Immunocytochemical localization of GPx5 protein was performed on epididymis tissue sections. There was no positive signal detectable along the epididymis in the lamb group (Figure 3A1–C1). However, much GPx5 protein was detected in the caput epithelial cells of young sheep. Some positive signals were observed in the corpus and cauda epithelial cells of young sheep (Figure 3A2–C2). In the epididymis of adult sheep, GPx5 protein was mainly expressed in the epithelial cells within the caput region. Low levels of GPx5 protein were expressed in the corpus and cauda regions (Figure 3A3–C3), but the signal was much fainter than that observed in the young sheep group. This is consistent with the expression pattern of GPx5 protein as described above. Additionally, the strongest positive signals were observed around the sperm in epididymis corpus and cauda (Figure 3).

To further investigate the location of GPx5 protein in the epididymis, the young sheep epididymis caput was divided into proximal (Figure 3D) and distal (Figure 3E) segments. From the proximal and distal caput epididymis segments, the GPx5 protein was expressed by the epithelial cells, and immunostaining was not found in the cell nucleus. Notably, the luminal sperm showed increasing GPx5 protein in the epididymis duct of the caput (distal) (Figure 3E). In the efferent duct (proximal), there were few spermatozoa (Figure 3D). There was also labelling in the epithelial cells and in the lumen of the caput.

## DISCUSSION

Sperm DNA damage is a major cause of male infertility in animals [16]. Sperm DNA damage is associated with preimplantation embryonic abnormal development resulting in miscarriage and abnormal offspring development [17]. Injecting DNA damaged spermatozoa into mouse oocytes not only affects embryo development, but also postnatal growth and behavior of the offspring [18]. There are many causes of sperm DNA damage, including sperm developmental defects, oxidative stress, and the lack of antioxidants *in vivo*. However, the most important factor is oxidative stress [19]. In the epididymis, a thiol-protamine disulfide crosslinking contributes to the packaging of sperm chromatin. Excess ROS disturbs chromatin assembly and the formation of disulfide bridges, leading to changes in sperm DNA integrity and cohesion state [20–22]. GPx5−/− mice showed that the cauda epididymal epithelium tissue suffered oxidative damage, as well as cauda-stored spermatozoa [13]. Therefore, GPx5 may play an important role in sperm maturation.

In this study, we examined the GPx5 transcription in the epididymis and testis of Small Tail Han sheep. Small Tail Han is a type of Mongolian sheep in the semi-humid agricultural areas of China. They are have early sexual maturity, and are precocious and highly prolific sheep which can breed during the whole year. The male duration of puberty is about 4 to 5 months, with sexual mature at approximately 7 to 8 months. The tissue-specific expression of GPx5 in male sheep epididymis and testis at different ages were investigated in our study.

The GPx5 transcription was detected in different segments of the epididymis and testis of sheep, and in the whole epididymis. However, the level of GPx5 transcription in the caput epididymis tissue was significantly higher than in corpus and cauda. This trend was especially pronounced in the young sheep group, but was hardly detected in the testis. Similar results were reported in rat, boar, mouse, bull, roe deer, and European Bison [10,12,23–26]. In our study, the GPx5 transcription level was higher in the corpus than in the cauda of Small Tail Han sheep. However, in European Bison, the level of GPx5 transcription in the cauda of the epididymis was higher than in the corpus [26]. Our results and those of other related studies have indicated the pattern of GPx5 transcription in the male reproductive duct may be species-specific.

Our study showed that GPx5 transcription was detected in lamb, but without detectable protein expression. This observation is particularly important considering that puberty occurs between 4 to 5 months in Small Tail Han sheep. In young sheep, the epididymis is fully developed and can secrete protein. In our study, GPx5 protein significantly increased during this period compared to that observed in the lamb group. These results are similar to those observed in the mice where the GPx5 gene was detected at 10 days postpartum, while the secreted protein was detected until the sperm entered the epididymis of the young sheep at 30 days postpartum [27]. A study of rat epididymis found that the GPx5 protein was detected at 44 days postpartum [10]. Therefore, the GPx5 protein that was secreted lagged behind the gene transcription in the epididymis. In this study, GPx5 levels were extremely decreased in the adult compared to the young sheep. It is possible that other GPx enzymes compensated for the decline of GPx5 protein during the development of the body’s antioxidant system. Some reports showed that the expression levels of GPx3 and snGPx4 mRNA in the GPx5−/− mice were higher than in the wide-type in the caput [13]. However, further studies are needed to confirm this hypothesis.

After birth, the entire epididymis will undergo drastic changes in cellular and structural composition before it completes development [28], as demonstrated by our results (Figure 3). During the development of the epididymis, epididymal epithelium with different cell types appear, including principal, basal cells, and clear cells. These cell types are primarily divided into the three structural regions of the epididymis (caput, corpus, and cauda). Immunohistochemistry showed the expression of GPx5 protein in the male sheep epididymis. The GPx5 protein was primarily secreted by epithelial cell cytoplasm in the caput epididymis. GPx5 was secreted to the epididymal lumen, where it binds to the transiting sperm and protects it from ROS damage. This result is similar to a series of studies in mice [12,29,30]. In our study, the proximal and distal sheep caput showed a positive signal, but the GPx5 protein signal in the distal was stronger than the proximal caput; a result which is also similar to a mouse study [9]. In a study of stallions and dogs, GPx5 protein was mainly present in the epididymis proximal [11]. These different results in different animal may indicate that the GPx5 protein distribution pattern in the epididymis is species-specific.

In a conclusion, the expression levels of GPx5 in sheep epididymis changed significantly across different segments and different age groups. Importantly, GPx5 protein is not detected in the epididymis of sheep before puberty. GPx5 was primarily expressed in the epithelial cells of the epididymis caput.

## Figures and Tables

**Table 1:** Real-time PCR cDNA amplification conditions

Gene	Oligonucleotides1)	Length (bp)	Annealing temperature (°C)
*Gpx5*	F:5′GCAATCCTGTCCTCACCCTT 3′ R:5′ACGCCATCAGGTCCCACT 3′	120	62
*β-actin*	F:5′GTCATCACCATCGGCAATGA3′ R:5′CGTGAATGCCGCAGGATT3′	88	62

PCR, polymerase chain reaction; GPx, glutathione peroxidase.

1) F: forward; R: reverse.
